# Supplementary material for: Stopping molecular rotation using coherent ultra-low-energy magnetic manipulations
Source: Nat Commun. 2022 Apr 28;13:2287. doi: 10.1038/s41467-022-29830-3 (PMC9050693; doi:10.1038/s41467-022-29830-3)
Supplement: Supplementary file 1 — Supplementary Information [file 41467_2022_29830_MOESM1_ESM.pdf]

Supplementary Information

Stopping a rotating D_2 molecule using coherent ultra-low-energy magnetic manipulations.

H. Chadwick et al.

Supplementary Note 1. Nuclear spin and rotational states of D_2 molecules

Individual D atoms have a nuclear spin, I_D, of 1, which means that the total nuclear spin of a D_2 molecule, I, can have values of 2, 1 or 0 depending on how the spins of the individual atoms couple. Molecules with even values of I correspond to ortho-D_2, and with odd values of I, para-D_2. As the D atoms are bosons, the molecular wave-function has to be symmetric with respect to interchange of the two nuclei, which restricts ortho-D_2 to having even rotational states, and para-D_2 to odd rotational states. Each I state has $2I + 1$ nuclear spin projection states (m_I), and each J state has $2J + 1$ rotational projection states (m_J). Supplementary Fig. 1 illustrates the relation between the total nuclear spin states and the allowed rotational states.

Supplementary Note 2. The Hamiltonian $\mathcal{H}_R(\boldsymbol{B})$

The Hamiltonian for D_2 in $J = 2$ interacting with a magnetic field can be written as[1–3]

$$\frac{\mathcal{H}_R(B)}{h} = -a\frac{\mathbf{I}\cdot\mathbf{B}}{\mathrm{B}} - b\frac{\mathbf{J}\cdot\mathbf{B}}{\mathrm{B}} + c\left(\frac{I(I+1)+J(J+1)-F(F+1)}{2}\right) - \tag{1}$$

$$15\sqrt{30}d(-1)^{F+I+J}(2J+1)\sqrt{(2I+1)(2I'+1)}\begin{pmatrix} J & 2 & J \\ 0 & 0 & 0 \end{pmatrix}\begin{Bmatrix} F & J & I' \\ 2 & I & J \end{Bmatrix}\begin{Bmatrix} 1 & 1 & 1 \\ 1 & 1 & 1 \\ I' & I & 2 \end{Bmatrix} +$$

$$\frac{2eqQ}{4h}(-1)^{2I+J+F+1}\sqrt{\frac{30(2I+1)(2I'+1)J(J+1)(2J+1)}{(2J-1)(2J+3)}}\begin{Bmatrix} 1 & I' & 1 \\ I & 1 & 2 \end{Bmatrix}\begin{Bmatrix} F & J & I' \\ 2 & I & J \end{Bmatrix}$$

where $\mathbf{F} = \mathbf{I} + \mathbf{J}$, a (653.6 Hz/gauss[3]) quantifies the interaction of the nuclear spin, **I** with the applied magnetic field, **B**, b (336.8 Hz/gauss[3]) the interaction of the rotational angular momentum **J** with the applied field, c (8723 Hz[1]) the spin-rotation interaction, d (2725 Hz[1]) the spin-spin interaction and $\frac{eqQ}{h}$ (223.38 kHz[1]) the quadrupole interaction of the two nuclei. It is these last two (field independent) terms which couple the $I = 2$ and $I = 0$ states of $J = 2$

together in the absence of a magnetic field. The expressions for the last three terms and the associated zero field energies of the states are given in Tables II and IV of reference 1 respectively.

Supplementary Note 3. Constraints on the S-matrix due to reflection symmetry

Assuming that the scattering process is achiral, the collision of the D_2 molecule with the Cu(111) surface should be symmetric with respect to the scattering (xz) plane[4]. As **J** behaves as a pseudovector under reflection, the x and z components of the vector change sign, but the y component remains unchanged[5]. This is shown schematically in Supplementary Fig. 3, where **J** is the red arrow.

The invariance of the distribution to reflection can be written as[4,5]

$$P(\theta,\phi) = RP(\theta,\phi) = P(\pi-\theta,\pi-\phi) \tag{2}$$

The rotational parts of the wavefunctions can be written in terms of the spherical harmonics, $C^J_{m_J}(\theta,\phi)$. The reflection in this case then gives $RC^J_{m_J}(\theta,\phi) = C^J_{m_J}(\pi-\theta,\pi-\phi)$. Using the properties of spherical harmonics that[6] $C^J_{m_J}(\theta,\phi) = (-1)^{m_J} C^J_{-m_J}(\theta,\phi)^*$ and[6] $(-1)^{J+m_J} C^J_{m_J}(\theta,\phi) = C^J_{m_J}(\pi-\theta,-\phi)^*$ leads to

$$C^J_{m_J}(\pi-\theta,\pi-\phi) = (-1)^J C^J_{-m_J}(\theta,\phi-\pi) \tag{3}$$

Using $\psi^J_{m_J} = C^J_{m_J}(\theta,\phi)$ for the (rotational part of the) wavefunction gives the following symmetry properties for the scattering matrix elements for achiral scattering.

$$S^{J'J}_{m_J' m_J} = \left\langle \psi^{J'}_{m_J'} \middle| S \middle| \psi^J_{m_J} \right\rangle$$

$$= \left\langle C^{J'}_{m_J'}(\theta,\phi)^* \middle| S \middle| C^J_{m_J}(\theta,\phi) \right\rangle$$

$$= \left\langle (-1)^{J'} C^{J'}_{-m_{J'}}(\theta, \phi - \pi)^* \middle| R^\dagger S R \middle| (-1)^J C^J_{-m_J}(\theta, \phi - \pi) \right\rangle$$

$$= (-1)^{J+J'} \left\langle C^J_{-m_J'}(\theta, \phi)^* e^{-i m_J' \pi} \middle| S \middle| C^J_{-m_J}(\theta, \phi) e^{i m_J \pi} \right\rangle$$

$$= \left(e^{i\pi}\right)^{J+J'} e^{-i m_J' \pi} e^{i m_J \pi} \left\langle C^J_{-m_J'}(\theta, \phi)^* \middle| S \middle| C^J_{-m_J}(\theta, \phi) \right\rangle \qquad (4)$$

$$= e^{i\pi(J + J' - m_{J'} + m_J)} S^{J'J}_{-m_J' - m_J}$$

For the case of scattering from J = 2 to J' = 0 considered here, it follows from Eq. (4) that $s_{02} = s_{0-2}$, $s_{01} = s_{0-1}$, $k_{02} = k_{0-2}$ and $k_{01} = k_{0-1} + \pi$. The S-matrix parameters were subject to these constraints during the fitting procedure.

**Parameter**	**Value**
# points in Z	180
# points in specular Z grid	384
Start of Z grids	-1.0 a_0
Step size of grids in Z	0.2 a_0
# points in r	64
Start of grid in r	0.4 a_0
Step size of grid in r	0.15 a_0
# points in X (Y)	24 (24)
Lattice constant	4.8764525 a_0
Maximum J value in basis set	14
Maximum m_j value in basis set	14
Time step for propagation	2.5 a.u.t.
Total propagation time	324300 a.u.t.
Location of analysis line in Z	9.2 a_0
Location of analysis line on specular grid Z	20.6 a_0
Location of the initial wavepaket	22 a_0
Normal incidence energy range	15 meV to 45 meV
Initial parallel incidence energy along [10-1] direction	7.3829 meV
Range of optical potential on Z grid	25.6 a_0
Target energy for optical potential on Z grid	2.5 meV
Range of optical potential on specular Z grid	40.8 a_0
Target energy for optical potential on specular Z grid	2.5 meV
Range of optical potential on r grid	4.0 a_0
Target energy for optical potential on r grid	100 meV
Mass of D atom	2.0156 u

Supplementary Table 1. The parameters used for the quantum dynamics calculations for D_2 scattering from Cu(111).

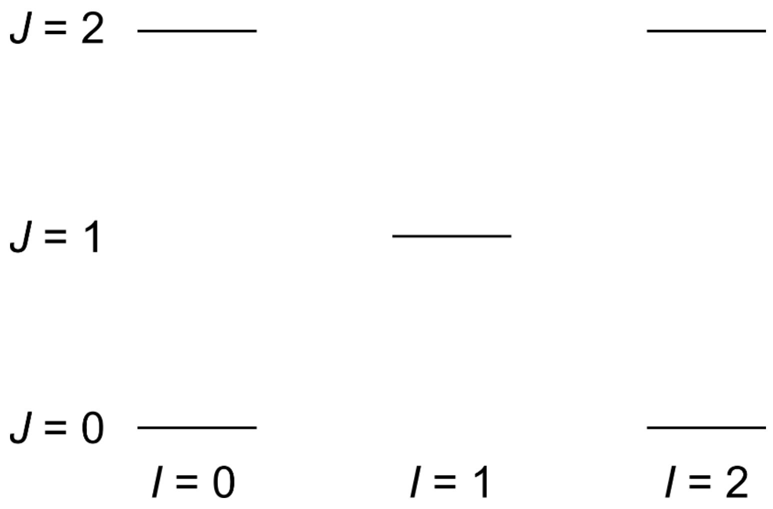

Supplementary Figure 1. Schematic representation of the nuclear spin states of D_2 and the lowest rotational states that they can have. The $I = 0$, $J = 0$ is a singlet, whereas $I = 0$, $J = 2$ splits into 5 non-degenerate states, $I = 1$, $J = 1$, into 9 non-degenerate states, $I = 2$, $J = 0$ into 5 non-degenerate states and $I = 2$, $J = 2$ into 25 non-degenerate states. The experiments we perform isolate the $J = 2 \rightarrow J = 0$ transition, and therefore involves 30 initial states, the field dependence of the eigen-energies is plotted in Fig. 3a of the main manuscript. Note that for a field of zero there are only 6 different eigen-energies, associated with 6 different hyperfine states[1].

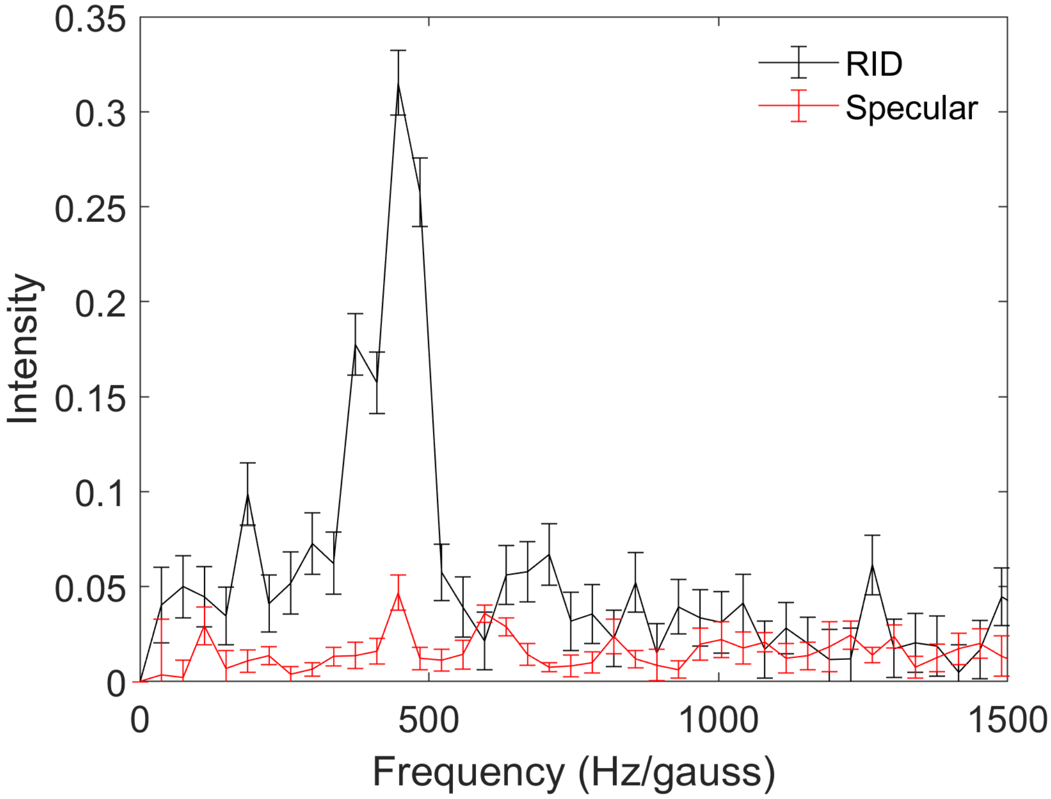

Supplementary Figure 2. Fourier transform of the $J = 2$ to $J = 0$ rotationally inelastic diffraction (RID, black) and the elastic specular (red) scattering data presented in Fig. 2c of the main manuscript showing the frequencies that contribute to the experimental data. The error bars represent the standard error of the measurements from repeated *B1* scans.

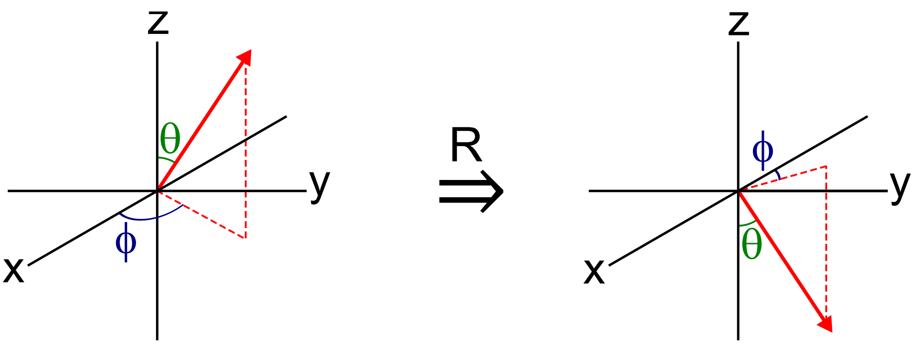

Supplementary Figure 3. Schematic representation of the effect of a reflection on a rotational angular momentum vector ***J***.

Supplementary References

1. Code, R. F. & Ramsey, N. F. Molecular-beam magnetic resonance studies of HD and D_2. *Phys. Rev. A* **4**, 1945–1959 (1971).
2. Bartlett, N. C.-M., Jankunas, J., Zare, R. N. & Harrison, J. A. Time-dependent depolarization of aligned D_2 caused by hyperfine coupling. *Phys. Chem. Chem. Phys.* **12**, 15689–15694 (2010).
3. Ramsey, N. F. Theory of molecular hydrogen and deuterium in magnetic fields. *Phys. Rev.* **85**, 60–65 (1952).
4. de Miranda, M. P., Aoiz, F. J., Bañares, L. & Rábanos, V. S. A unified quantal and classical description of the stereodynamics of elementary chemical reactions: State-resolved k–k′–j′ vector correlation for the H+D_2(v=0, j=0) reaction. *J. Chem. Phys.* **111**, 5368–5383 (1999).
5. Aoiz, F. J., Brouard, M. & Enriquez, P. A. Product rotational polarization in photon-initiated bimolecular reactions. *J. Chem. Phys.* **105**, 4964–4982 (1996).
6. Zare, R. N. *Angular momentum understanding spatial aspects in chemistry and physics*. (Wiley, 1988).
